# An overview and thematic analysis of research on cities and the COVID-19 pandemic: Toward just, resilient, and sustainable urban planning and design

**DOI:** 10.1016/j.isci.2022.105297

**Published:** 2022-10-07

**Authors:** Ayyoob Sharifi

**Affiliations:** 1 Hiroshima University, Graduate School of Humanities and Social Science, Higashi-Hiroshima, Hiroshima, Japan; 2 Network for Education and Research on Peace and Sustainability (NERPS); 3 Center for Peaceful and Sustainable Futures (CEPEAS), The IDEC Institute, Hiroshima University

**Keywords:** Environmental issues, urban planning, human geography

## Abstract

Since early 2020, researchers have made efforts to study various issues related to cities and the pandemic. Despite the wealth of research on this topic, there are only a few review articles that explore multiple issues related to it. This is partly because of the rapid pace of publications that makes systematic literature review challenging. To address this issue, in the present study, we rely on bibliometric analysis techniques to gain an overview of the knowledge structure and map key themes and trends of research on cities and the pandemic. Results of the analysis of 2,799 articles show that research mainly focuses on six broad themes: air quality, meteorological factors, built environment factors, transportation, socio-economic disparities, and smart cities, with the first three being dominant. Based on the findings, we discuss major lessons that can be learned from the pandemic and highlight key areas that need further research.

## Highlights

• We provide an overview and thematic analysis of research on cities and COVID-19
• We identify six thematic areas, including air quality and environmental factors
• We highlight some under-explored areas that need to be further studied in the future
• We discuss issues for guiding a more just, resilient, and sustainable recovery

## Graphical abstract

**Figure:**
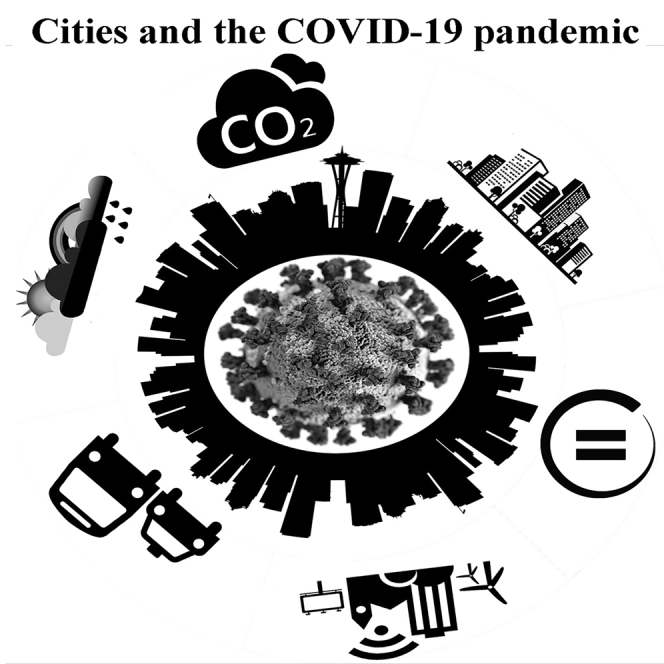

## Introduction

The global population has rapidly urbanized since the middle of the twentieth century. The world urban population is currently over 4.2 billion, up from 751 million in 1950. Urbanization trends are projected to continue to increase in many parts of the world in the coming decades. According to the UN projections, rapid urbanization trends will continue in many parts of the world. By 2050, the population living in cities will be about 6.68 billion inhabitants, reaching a record high level and accounting for about 68 percent of the world’s population.

Because of these trends and the major socio-economic and environmental implications of urbanization, cities have increasingly gained the attention of researchers and policy-makers in the past few decades. The COVID-19 pandemic emerged amidst these developments and added new momentum to research focused on cities. Historically, cities have been epicenters of pandemics and epidemics, and public health crises have played major roles in the evolution of urban planning and design ideas and techniques (La Greca et al., 2020). Despite this, a literature search in academic databases such as the Web of Science (WoS) shows that issues at the interface of cities and pandemics were not well studied before the emergence of the COVID-19 pandemic. Since 2020, however, many researchers have endeavored to shed light on various issues related to cities and pandemics. As will be further discussed in the next section, about three thousand papers related to this issue have been indexed in the WoS in 2020 and 2021, and more are expected to be published in the next few years. Existing studies have improved our knowledge of the transmission and control dynamics of the pandemic and have allowed reflection on commonly practiced urban planning, design, and management principles to develop transformative solutions for a more green, inclusive, and resilient recovery (Sharifi and Khavarian-Garmsir, 2020; Alidadi and Sharifi, 2022).

The wealth of studies on cities and the COVID-19 pandemic has provided an opportunity to conduct reviews that synthesize the reported findings and provide recommendations for developing better planning, response, and control mechanisms. Accordingly, review studies have been published on various specific topics such as air quality changes (Adam et al., 2021; Rana et al., 2021; Faridi et al., 2021; Gope et al., 2021), impacts of weather and meteorological factors on the infection risk (Sharma et al., 2021; Zhu et al., 2021), monitoring the transmission of the virus in the urban water systems (Pena-Guzman et al., 2021; Sunkari et al., 2021), green recovery in cities (Moglia et al., 2021), contributions of smart city solutions and technologies to pandemic control (Sharifi et al., 2021), associations between built environment factors and the infection risk (Wang et al., 2021), impacts on urban mobility and transport (Kakderi et al., 2021; Rojas-Rueda and Morales-Zamora, 2021; Gkiotsalitis and Cats, 2021; Ceder, 2020), responding to the needs of the homeless (Jang et al., 2021), housing (Kaklauskas et al., 2021), impacts of urban environment on mental health (Menculini et al., 2021), the role of greening and nature-based solutions (Bayulken et al., 2021), urban health (Alhassan et al., 2021), urban agriculture (Lal, 2020), and context-specific policy responses for building greener and pandemic-proof cities (Angiello, 2020; Gaglione, 2020). These studies have highlighted some problems that cities have been facing for a long time in a new light and have offered solutions to address them.

However, despite their contributions, existing reviews tend to focus on only one or a few urban sectors. This is unsurprising given the volume of research published and the rapid pace of publication that makes it challenging to cover multiple urban sectors in a single review article. Nonetheless, covering multiple sectors is desirable as it allows understanding intersectoral interlinkages. In addition, a multisectoral focus is conducive to identifying understudied sectors that deserve further attention. One way to partially address this issue is to use bibliometrics that provides tools for understanding the knowledge structure and trends of rapidly growing research fields (Cobo et al., 2011a). Bibliometrics can be used for science mapping or performance analysis. Science mapping offers information on the thematic structure of a research field and its evolution over time and helps unpack interlinkages between different themes and sub-themes. Performance analysis complements science mapping by identifying influential authors, references, journals, and institutions/countries that have contributed more to the development of a research field (Cobo et al., 2011a). Some bibliometric analyses on issues related to the pandemic have been conducted (Benita, 2021). However, there is a lack of a bibliometric analysis that examines multiple issues.

Against this background, this article aims to provide an overview of the research published on the COVID-19 pandemic and cities in 2020 and 2021. Specific objectives are to identify major thematic areas, discuss how they have changed over time, and highlight influential references, journals, and authors. The article also highlights key lessons learned from the pandemic and offers some recommendations regarding thematic areas that are relatively under-explored and warrant further research. It should be noted that this article is different from systematic reviews that offer detailed syntheses of specific issues related to cities and the pandemic. It complements such review studies by providing a broader overview, highlighting thematic interlinkages, and identifying major themes, references, journals, and authors. Results can be used to better understand the current structure of knowledge related to the topic, highlight key resources (i.e., journals and references) that interested researchers and policy-makers can refer to for gaining more information and identify potential research gaps.

## Results and discussions

### Overview

Overall, 2,799 articles related to the topic were selected for analysis after careful screening. Regarding the document type, about 94% are research articles, about 5% are review articles, and the rest are letters, data papers, and proceedings papers. In terms of the publication date, 813 (∼29%) have been published in 2020 and 1,986 (∼71%) in 2021. This is a clear indication of the great interest shown by researchers in analyzing issues related to the pandemic and cities. As expected, the published articles have also been highly cited (over 26,000 times). This is likely to continue in the coming years owing to the following reasons: 1- the pandemic is still a long way from being fully controlled, and new waves offer additional data and opportunities for further analysis; 2- more time is needed to examine some socio-economic impacts of the pandemic that may take longer time to appear; and 3- pandemic and publication of research outputs could take several months or years in some cases. As shown in Table 1, publications on this topic cover various research areas, including environmental science, engineering, business economics, and transportation. Major thematic focus areas will be further discussed in the remainder of this section. Table 1 also shows that the US, China, and Italy are the main contributing countries.

**Table 1:** Major research areas of the analyzed articles and the main contributing countries (Source: Web of Science)

Research areas (based on Web of Science)	% of total	Country	% of total
Environmental Sciences Ecology	36.299	USA	24.151
Science Technology Other Topics	17.828	China	17.256
Public Environmental Occupational Health	16.184	Italy	9.218
Urban Studies	8.789	England	9.003
Business Economics	6.074	India	7.788
Geography	6.038	Spain	5.431
Transportation	5.931	Canada	5.002
Engineering	5.502	Brazil	4.895
Public Administration	4.394	Australia	4.359
Meteorology Atmospheric Sciences	4.001	Germany	3.287

### Core research themes and their change over time

The term co-occurrence analysis in VOSviewer was used to identify research topics and themes that have received more attention. In addition to analyzing all articles, we did separate analyses of papers published in 2020 and 2021 to examine changes in thematic focus over time.

**Figure 1:**
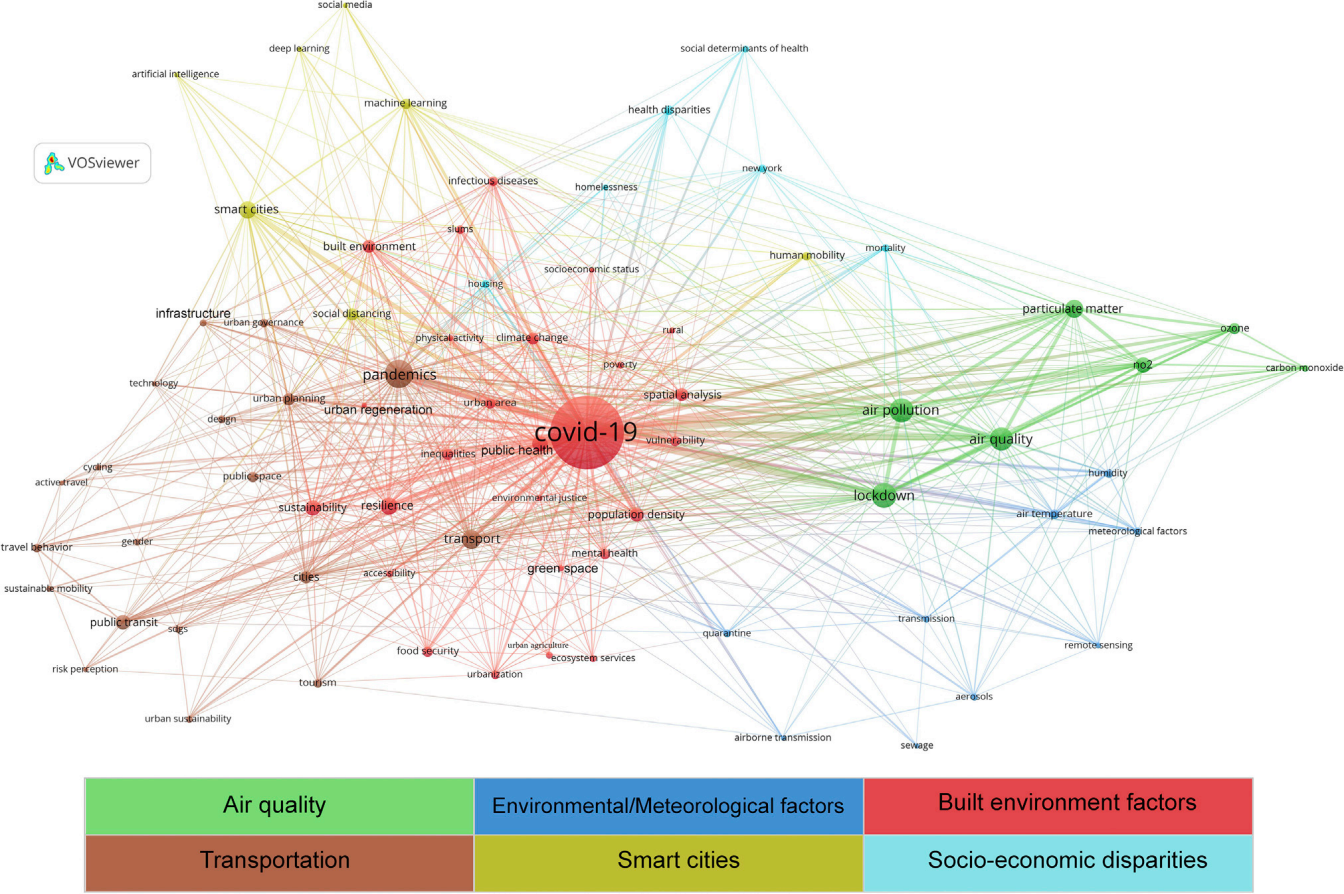
Major thematic focus areas of the research published on cities and the COVID-19 pandemic

The output of the term co-occurrence analysis is shown in Figure 1. As mentioned earlier, the relative dominance of a theme could be determined based on the node size and the width of the links connecting the nodes. Six major thematic areas could be distinguished in Figure 1: air quality, meteorological factors, built environment factors, transportation, inequalities, and socio-economic disparities, and smart city solutions and technologies. To a large extent, these are consistent with the following thematic categories listed in a systematic review paper published at the end of 2020 by Sharifi and Khavarian-Garmsir (2020): Environmental quality, socio-economic impacts, management and governance, and transportation and urban design.

#### Air quality

Terms related to air quality are dominant (the green cluster), indicating that much research has been published on the impacts of the pandemic and associated mobility restrictions on air quality. This includes impacts on different types of pollutants, such as Particulate Matter 2.5 (PM_2.5_), Particulate Matter 10 (PM_10_), Carbon Monoxide (CO), Nitrogen Dioxide (NO_2_), Sulfur Dioxide (SO_2_), and Ozone (O_3_), and impacts on anthropogenic heating in cities (Silva et al., 2022; Shen et al., 2021; Ravina et al., 2021; Roshan et al., 2022; Jiang et al., 2021). Researchers have compared levels of pollutant concentration before and during the pandemic, finding varying impacts depending on the context and type of pollutants (Silva et al., 2022; Sharifi and Khavarian-Garmsir, 2020). In most cases, NO_2_ and CO, pollutants closely associated with urban transport, have decreased in big cities worldwide, especially in major polluted cities of countries like China and India (Sharifi and Khavarian-Garmsir, 2020; Baldasano, 2020; Sharma et al., 2020; Zhao et al., 2022). This indicates that major air quality improvements can be achieved by greening the transportation sector (Sharifi and Khavarian-Garmsir, 2020). However, focusing on the transportation sector would not be enough as the mobility restrictions had not always resulted in the reduction of other types of pollutants like PM_2.5_, PM_10_ (Menut et al., 2020; Sharifi and Khavarian-Garmsir, 2020). Although in some countries like India significant reductions in the concentration of particular matter were observed during the lockdown periods (Roy and Singha, 2021), no such reductions have been reported in other parts of the world like some Western European Cities (Menut et al., 2020) or some cities in China (Nichol et al., 2020; Ethan et al., 2021). This indicates that, in some contexts, other factors and sources such as industrial activities, biomass burning, domestic heating, and long-distance transport of particles are major contributors to particular matter concentration (Dantas et al., 2020; Sharifi and Khavarian-Garmsir, 2020). Therefore, context-specific measures that consider such sources are important for urban air quality improvement in the post-COVID era.

Figure 1 shows that the terms “air quality” and “air pollution” are closely linked to “transmission.” In fact, the impacts of air pollution on the transmission dynamics and vulnerability to COVID-19 have been extensively explored in the literature. Evidence shows that transmission rates are higher in polluted areas (Fernandez et al., 2021; Tian et al., 2021; Coccia, 2020). Furthermore, through detrimental effects on the respiratory system, air pollution could increase vulnerability to infectious diseases such as COVID-19 and lead to higher mortality rates (Zhu et al., 2020; Tian et al., 2021; Sharifi and Khavarian-Garmsir, 2020). Accordingly, prioritizing air quality improvements can ensure better resilience to future pandemics by facilitating enhanced control mechanisms and improving the coping capacity of urban residents (Sharifi and Khavarian-Garmsir, 2020).

It is worth noting that despite the wealth of research on the air quality impacts of the pandemic, there are issues that need to be further studied in future research. More research is needed to understand changes in the concentration levels of CO_2_ and CH_4_ in cities during the lockdown periods. This can provide useful insights for the transition toward climate change mitigation in cities. The lack of longitudinal studies on air pollution is another key issue that needs to be addressed to find out how the conditions have changed after returning to normal and if the lessons offered by the pandemic have been used to enhance the air quality in cities. Also, more research is needed to estimate the health and economic co-benefits that air quality enhancements during the pandemic have provided. For instance, this could include the estimation of the avoided premature deaths and productivity improvements.

#### Meteorological factors

Closely related to the green cluster, the blue cluster is focused on environmental and meteorological factors and their impacts on the transmission of COVID-19. As can be seen from the blue cluster in Figure 1, temperature and humidity are two meteorological factors that have received more attention. As the impacts of these factors have been examined in different cities around the world, and given the influences of other confounding factors, the reported results are mixed. For instance, examining associations between average air temperature and relative humidity and the daily number of new COVID-19 cases in nine cities in Asia, He et al. (2021) found positive associations in most cases, but not in all. Similar positive associations have also been reported in Norway (Menebo, 2020), Italy (Zoran et al., 2020), and Iran (Jahangiri et al., 2020). In contrast, negative or no associations between meteorological factors (temperature and humidity) and the spread of the virus have been reported in studies examining cases in China (Lin et al., 2020) and different areas of South America and Africa (Babuna et al., 2021). This mixed and inconclusive evidence also shows that contextual factors and confounding factors such as population density, lockdown policies, human behavior, and differences in health infrastructure may affect the nature of the association between meteorological factors and the spread of COVID-19 (Diao et al., 2021; Sharifi and Khavarian-Garmsir, 2020). Wind speed is another meteorological factor that has been studied in the literature but does not appear in Figure 1. This indicates that evidence on the effects of wind speed on transmission patterns in cities is relatively limited. Some studies of Italian cities have argued that, by decreasing the concentration of urban air pollutants that could be an indirect means of virus spread, higher wind speed lowers the number of COVID-19 cases (Coccia, 2021; Zoran et al., 2020). However, such associations have not been confirmed in studies examining cities in Turkey (Şahin, 2020) and China (Lin et al., 2020). Overall, more research on this issue is needed.

Another noteworthy term at the bottom of the blue cluster is sewage. Given the transmission of COVID-19 through the urban water cycle and fecal-oral routes (Ul Haque et al., 2021), adequate provision and management of urban sewage systems are essential for controlling the pandemic. There is a need for proper measures to minimize water pollution at the point source and prevent exposure to sewage and its leakage into freshwater resources (Naddeo and Liu, 2020; Bhowmick et al., 2020). This is particularly important in some cities in the Global South that lack adequate water management and sewage systems. Another issue related to the sewage system that has been mentioned in the literature is the possibility of using sewage monitoring systems to investigate the distribution of the virus and identify hotspots (Mota et al., 2021). Such monitoring applications can serve as early warning systems that enhance cities' prediction and response capacities. Integration of such monitoring systems into the urban infrastructure system, particularly in the Global South cities, could be challenging owing to technical feasibility issues, lack of skilled personnel, potentially high costs, and maintenance requirements.

#### Built environment factors

The red cluster in the middle of Figure 1 mainly focuses on urbanization and built environment factors that could influence the spread of the virus and/or the capacity to control the pandemic. The key terms of this cluster are closely linked to terms from other thematic clusters. This is because urban issues are at the center of this bibliometric analysis and, as mentioned earlier, built environment factors such as density could influence how other factors affect pandemic transmission and control dynamics. Population density is a key term in this cluster. Many studies have examined the associations between different indicators of density and COVID-19 cases and mortality (Hamidi et al., 2020; Lak et al., 2021a, 2021b). This is unsurprising as, urban planners have always been interested in examining the impacts of density on various urban issues (Sharifi, 2019), and historically, there have been concerns over density being a risk factor for the spread of infectious diseases (Fezi, 2020). Also, some historical evidence indicates higher infectious disease mortality in denser areas (Fezi, 2020). Despite this, evidence on the associations between density and COVID-19 cases and mortality is mixed and inconclusive. Results reported for cities in Chile (Rivera-Cordova, 2021), China (You et al., 2020), and Iran (Lak et al., 2021b) indicate positive associations. However, either no or negative associations have been found in many other studies (Boterman, 2020; Federgruen and Naha, 2021; Khavarian-Garmsir et al., 2021). Overall, the prevailing argument is that physical factors such as density and city size, per se, do not increase the risk of infection (Sharifi and Khavarian-Garmsir, 2020). In fact, governance mechanisms and other mediating factors such as socio-economic factors, the degree of connectivity (local, regional, and international), the level of compliance with COVID-19 control measures, quality and access to infrastructure, and socio-economic factors like income and human behavior are important and should be considered (Habitat, 2021; Chu et al., 2021; Liu et al., 2021). As the figure shows, terms related to socio-economic status and justice are highlighted in this cluster and are closely linked to build environment factors. These are highly connected to the light blue cluser on inequalities and socio-economic disparities and will be further discussed later.

Terms related to parks and urban greenery are highlighted at the bottom of the red cluster. Different aspects of parks and urban greenery have been investigated in the literature. The need for ample and equitable distribution of green spaces in urban areas has long been recognized. The pandemic has further increased interest in such spaces, as shown in a global analysis of urban park visitation trends before and during the pandemic in many countries around the world (Geng et al., 2021). The mobility restrictions imposed during the pandemic increased the risk of alienation and associated stress and anxiety issues (Pouso et al., 2021; Cheng et al., 2021). Under such circumstances, access, and visiting parks and green spaces have helped people of different ages maintain their mental and physical health (Cheng et al., 2021; Levinger et al., 2021; Pouso et al., 2021). However, this increased interest has exposed issues related to the lack of accessibility and inequitable distribution of parks and green spaces in a new light (de Lannoy et al., 2020). Accordingly, appropriate policies and design measures are needed to enhance environmental justice by improving access for all societal groups (Levinger et al., 2021; Scott, 2021). Urban agriculture is a specific form of urban greenery that provides multiple ecosystem services and has gained new momentum over the past two years. An important issue highlighted by the pandemic was the vulnerability of cities to supply chain disruptions, particularly those related to the food supply. Disruptions were mainly caused by mobility restrictions that prevented food transport from farm to markets or by international trade restrictions made to ensure meeting the domestic supply of food. Such disruptions have particularly affected low-income groups that could not afford the increased prices (also those who become unemployed) (Blay-Palmer et al., 2021). Although local food production is not a panacea, it contributes to enhancing food security by diversifying the food supply structure and shortening supply routes (Blay-Palmer et al., 2021). Therefore, finding a balance between global connectivity and local self-sufficiency is critical for improving urban resilience to future threats (Habitat, 2021). During the pandemic, urban agriculture and improved urban-rural linkages that facilitated direct connections between farmers and consumers have played a vital role in ensuring the food security of low-income vulnerable groups in many cities worldwide (Blay-Palmer et al., 2021). For instance, urban agriculture and access to allotment gardens contributed to mitigating the food security impacts of the COVID-19 pandemic in Benin (Houessou et al., 2021). Similarly, local food production has contributed to food security in communities across South Africa, Mozambique, Zimbabwe, and Indonesia (Paganini et al., 2020). Overall, further investment in urban agriculture in the post-COVID era is needed for enhancing urban resilience and reducing inequalities.

#### Inequalities and socioeconomic disparities

In addition to inequitable access to green spaces and ecosystem services, vulnerabilities associated with poverty and health disparities have also received considerable attention, as can be seen in the light blue cluster (Figure 1). Evidence from different contexts shows higher rates of morbidity and mortality among racial and ethnic minorities, migrants, and low-income groups (Mishra et al., 2021; Vilar-Compte et al., 2022; Creţan and Light, 2020). Several factors have contributed to such differential impacts. An important factor is a social vulnerability and the lack of access to health and sanitation infrastructure (Bin Kashem et al., 2021). For instance, inequitable provision and distribution of infrastructure in some North American cities have been linked to higher pandemic vulnerability among some race-based and low-income communities (Enright and Ward, 2021). Other noteworthy factors are precarious livelihoods that could result in ignoring stay-at-home orders; and unfavorable living conditions (e.g., living in crowded slums) that could make it challenging to comply with hygiene and social distancing measures (Sharifi and Khavarian-Garmsir, 2020; De Groot and Lemanski, 2021; Wrigley-Field et al., 2021; Alizadeh and Sharifi, 2022).

Overall, vulnerable groups have been disproportionately impacted by the pandemic and impacts caused by periods of recession and increasing unemployment (Creţan and Light, 2020; Sharifi and Khavarian-Garmsir, 2020). In fact, there are arguments that the pandemic has amplified the existing urban inequalities (Turok and Visagie, 2021). An important issue is that, in some cases, inappropriate measures taken to respond to the pandemic have contributed to this. For instance, research in South Africa, Mozambique, Zimbabwe, and Indonesia shows that COVID-19 response measures have worsened socio-economic inequalities among some groups, leading to food insecurity issues (Paganini et al., 2020). It is also discussed that the urgent introduction of telemedicine programs in the US has increased healthcare disparities among minorities (Gmunder et al., 2021).

While vulnerable groups are disproportionately affected by the pandemic, a major lesson that can be learned is that presence of inequalities in a society is a barrier to effective response and control measures and could also undermine the safety of advantaged groups (Moglia et al., 2021). Accordingly, empowering vulnerable and marginalized groups and ensuring equitable access to infrastructure and services should be prioritized for inclusive post-COVID recovery (Moglia et al., 2021). This could, however, be challenging given the long-standing structural inequalities observable in many cities and the fact that about 30% of the global urban population is currently living in slums (UN, 2018). As emphasized in the Agenda 2030, global partnership and cooperation would be essential for this purpose (UNSDG, 2015).

#### Transportation

Issues related to transportation (brown cluster) have also been widely studied, as shown in Figure 1. This is unsurprising as concerns over the role of population movement in the spread of the virus and subsequent mobility restrictions have led to major impacts on the transportation sector (Wu et al., 2020). The figure shows that public transit has frequently been studied in the literature. Concerns over the higher risk of transmission in public transit systems have led to record declines in public transit ridership and network closures in some cities (Enright and Ward, 2021). In turn, the finance available for the maintenance and expansion of public transport systems has also shrunk in some cities (Restrepo, 2021; Enright and Ward, 2021).

Two major contrasting travel behavior effects have been observed following the decline in public transit ridership. The first one is an increase in private car use, as reported in some countries like India (Thombre and Agarwal, 2021). This could exacerbate congestion, air pollution, and traffic accidents, undermine climate change mitigation efforts, and delay the successful recovery of the urban economy (Hörcher et al., 2021). Therefore, it requires the due attention of planners and policy-makers. People make their travel choices based on different criteria such as time, cost, convenience, safety, and reliability. In the future, and as environmental awareness raises, other factors such as being environmentally friendly may also be prioritized. Therefore, to facilitate a mode shift from private cars to public transportation, conditions associated with such criteria should be improved (Fezi, 2020). Effective recovery of public transit systems also hinges on the ability to ensure social distancing. It is suggested that a combination of different measures such as “(i) inflow control with queueing, (ii) time and space dependent pricing, (iii) capacity reservation with advance booking, (iv) slot auctioning, and (v) tradeable travel permit schemes” could help achieve this goal. However, implementation of some of these measures may require adequate availability and accessibility to data and technological infrastructure and skilled personnel to ensure real-time data processing for informed decision-making (Hörcher et al., 2021). The second effect that has received more attention in the literature is the shift toward active transportation, including walking and cycling (Scorrano and Danielis, 2021; Shaer and Haghshenas, 2021; Buchel et al., 2022). These shifts and the tangible environmental quality benefits of large-scale traffic reductions, which were discussed earlier, have provided unprecedented opportunities to redesign the streetscape to reallocate underutilized public spaces for cycling lanes and pedestrian space. Such reconfigurations have already taken place in cities such as Barcelona, New York, and Portland-Oregon (Devine-Wright et al., 2020; Bojovic et al., 2020). Providing more space for active transportation also helps prevent the overloading of public transport systems, thereby ensuring better resilience to future pandemics (Barbarossa, 2020). Such efforts to promote active transportation would also contribute to decarbonizing urban transport and meeting urban climate change mitigation targets. These contributions could be maximized by adopting integrated approaches that include other elements such as green infrastructure in streetscape design to ensure co-benefits. For instance, the cycling and pedestrian corridors should be integrated with urban green infrastructure networks to make the environment more appealing and provide health/adaptation co-benefits (Valente et al., 2021).

#### Smart city solutions and technologies

The last cluster of smart cities (yellow color) is closely linked to the transportation cluster. Applications of smart city solutions and technologies could offer opportunities to build on these transformations and sustain the momentum. For instance, teleworking can complement measures aimed at reducing travel demand since some work trips that cannot easily be made through active modes will be canceled (Bojovic et al., 2020). Automation could also play a key role; autonomous vehicles (particularly public autonomous vehicles such as autonomous buses) are likely to reduce the need for private cars and further promote public transit and shared mobility (Ceder, 2020; Mouratidis et al., 2021). This could happen as, through being coupled with car sharing and mobility-*as*-a-service schemes, autonomous vehicles can improve the accessibility of different social groups and offer mobility services at lower costs and with higher comfort (Duarte and Ratti, 2018; Mouratidis et al., 2021). Regarding shared mobility schemes, bike-sharing is a mode that has been widely studied in the literature (Kim et al., 2021; Hu et al., 2021). Evidence from cities like New York City indicates that, unlike subway ridership, bike-sharing ridership has returned to pre-pandemic levels soon after the lockdown periods, indicating it is a resilient public transportation mode (Wang and Noland, 2021). Bike-sharing systems that are appropriately integrated into the public transit system could also be an alternative solution for the last-mile connection problem, thereby contributing to further reduction of automobile use (Pase et al., 2020).

Smart city solutions and technologies such as machine learning and artificial intelligence have also been discussed in relation to other sectors beyond transportation. They have shown great potential in enhancing cities’ capacity to prepare for, recover from, and adapt to the impacts of the pandemic. Such solutions and technologies have been utilized to predict the transmission patterns, trace and track infected individuals, maintain city operations during lockdown periods, reduce supply chain disruptions, and facilitate optimized and integrated urban governance and management (Sharifi et al., 2021; Leng et al., 2021). For instance, to address issues related to disruptions in the food supply, in some countries like South Korea, web-based trading platforms have been used to create direct links between consumers and farmers (Blay-Palmer et al., 2021). Urban observatories have also been used in different contexts to facilitate timely response to changing demands, provide opportunities for stakeholder engagement, reduce sectoral conflicts, and address interactions between different sectors across different scales through integrated multi-level governance systems (Moglia et al., 2021). Despite these benefits, there have been some concerns regarding data privacy or the spread of misinformation on social media (Sharifi et al., 2021). Those, however, do not appear in Figure 1, indicating the need for more research that, for example, demonstrates if and how smart solutions and technologies (e.g., based on artificial intelligence, internet of things, and machine learning techniques) can be utilized to address issues of privacy and data security that matter to urban residents, promote more sustainable urban development patterns through reforming urban economic structure, facilitate integrated urban management, and enhance planning, absorption, recovery, and adaptation capacities in the face of adverse events.

#### Changes over time

**Figure 2:**
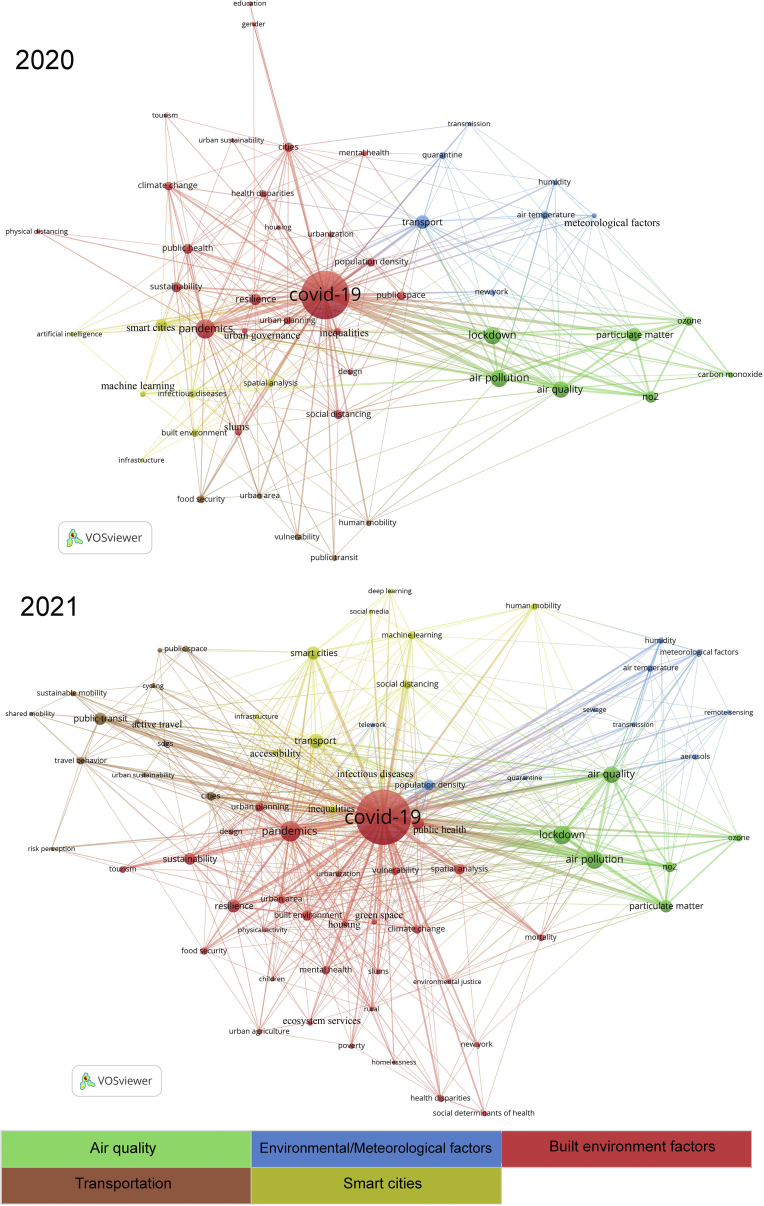
The term co-occurrence analysis results for 2020 (top) and 2021 (bottom)

Figure 2 shows the results of the term co-occurrence analysis for the documents published in 2020 and 2021. Results show that the core themes have remained the same in both years and are the same as those discussed in the previous sections. The only difference is that inequalities and socio-economic disparities are part of the theme of urban planning and built environment factors here. These two were also very closely linked in Figure 1. Air quality and environmental/meteorological factors are key themes in both years, and their structures remain almost the same. However, a closer look at the figure reveals some changes. The term “sewage” appeared in 2021, indicating that issues related to the sewage systems and their role in the transmission of the virus were mainly examined in 2021. Regarding the built environment factors (red cluster), it can be seen that there has been an emphasis on spatial analysis of the COVID-19 spread and socio-economic disparities in both years. However, terms such as green space, ecosystem services, urban agriculture, and food security only appear in 2021. This shows that researchers have initially just concentrated on factors affecting morbidity and mortality patterns and air pollution that are the immediate impacts of the pandemic, and other issues received attention later when more data on other impacts have become available. Another term that only appears in 2021 is “rural,” indicating that, over time, researchers have paid attention to urban-rural disparities and interactions (Visagie and Turok, 2021), and the need for multi-level governance systems that consider interactions between urban, peri-urban, and rural settlements (Spencer et al., 2021). Such governance systems are important for preventing unregulated urbanization patterns and minimizing encroachment on natural habitats that may increase infectious diseases, as evidence from some Asian countries shows (Spencer et al., 2021). Considering urban-rural linkages is also critical for minimizing food supply chain disruptions during adverse events like pandemics (Blay-Palmer et al., 2021). Despite these, the term “rural” has a marginal position in the output maps and warrants further research to better understand the implications of the pandemic for dynamic interlinkages across the urban-rural interface, the importance of integrated planning processes that recognize the interconnectivity and interdependency of urban and rural domains, and the governance and regulatory systems required to minimize possible encroachment on natural resources in peri-urban areas owing to pandemic-induced outmigration from large cities.

Structures of the smart cities clusters are similar in both periods, with larger node sizes in the latter. This is in line with the arguments that the pandemic has given additional momentum to research on smart cities (Sharifi et al., 2021). Transportation-related research is another theme that has significantly expanded over time. In the first period, the term “public tarnsport” is represented by a small node with few connections to the other terms. However, its position strengthened in 2021. It is linked to different transportation-related terms and issues such as travel behavior, active travel, cycling, shared mobility, sustainable mobility, and public space that were discussed in detail earlier. Obviously, the pandemic has been influential in stimulating discussions on transition toward sustainable mobility.

### Influential references

**Figure 3:**
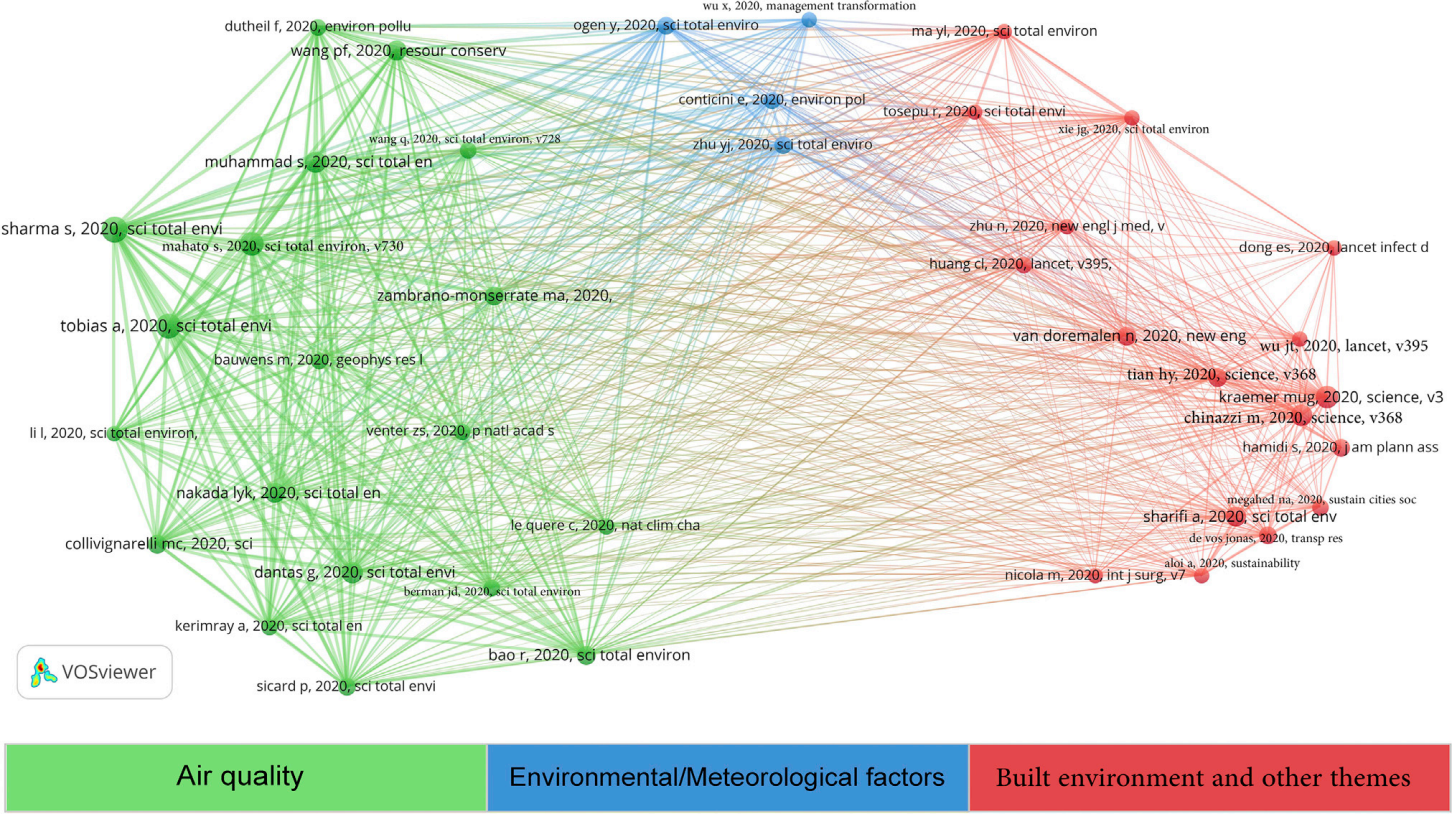
The most influential documents

The co-citation analysis was used to identify the most influential publications. Results are shown in Figure 3. Detailed quantitative data related to this analysis are available in the Online supplementary information (Table S1).

Three major clusters can be identified in Figure 3. The green cluster includes articles focused on air quality changes in urban areas during the pandemic. As can be seen, this is the dominant cluster with the most highly cited articles. This is unsurprising as it was earlier discussed that air quality has been a dominant research theme in 2020 and 2021. Noteworthy articles from this cluster include Sharma et al. (2020), Tobías et al. (2020), and Mahato et al. (2020).

The blue cluster includes articles related to the impacts of air pollution on the transmission of COVID-19. Articles in this cluster are connected to the other two clusters, indicating that they have also addressed issues related to air quality changes and other factors that could have influenced the spread of the virus. Influential articles from this category are Zhu et al. (2020), Ogen (2020), and Conticini et al. (2020).

The red cluster is composed of articles related to different themes. Articles in the top corner of the cluster are focused on the impacts of temperature and humidity on the spread of the virus. These include Ma et al. (2020), Tosepu et al. (2020), and Xie and Zhu (2020). Articles in the middle of the cluster address issues related to the effects of human mobility and travel restrictions on the spread of the pandemic. These include, for instance, Kraemer et al. (2020), Chinazzi et al. (2020), and Tian et al. (2020). Other highly influential references in this cluster are focused on major lessons of the pandemic for urban planning and design (Hamidi et al., 2020; Megahed and Ghoneim, 2020; Sharifi and Khavarian-Garmsir, 2020), effects on urban transportation and travel behavior (De Vos, 2020; Aloi et al., 2020), socio-economic implications of the pandemic (Nicola et al., 2020), and smart city solutions (Dong et al., 2020).

These results are in line with the results of the thematic analysis and indicate the dominance of research focused on air quality, environmental/meteorological factors, and built environment and human mobility factors in the literature. In other words, issues related to smart cities and socio-economic disparities are relatively under-explored. An important issue that can be noted from this analysis is the dominance of articles published in the first three-quarters of 2020. In addition to their scientific importance, it is likely that being published during the first months of the pandemic has allowed them to get more publicity and recognition. This has helped these articles amass relatively more citations over time and this is likely to continue in the coming years. As a result, only reliance on this list would not be sufficient and it should be acknowledged that other informative recent references exist that might not have many citations. Therefore, researchers should also consider more recently published articles when designing their studies.

### Influential journals, authors, and countries

**Figure 4:**
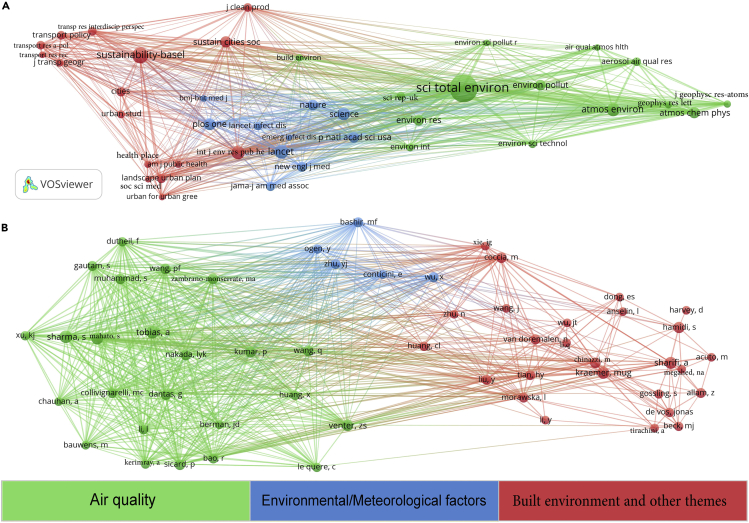
The most influential journals (A), and authors (B).

Co-citation analyses were also conducted to identify prominent journals and authors (setting sources and authors as units of analysis). Results are shown in Figure 4. Also, detailed quantitative information related to these analyses can be found in Tables S2 and S3 of the supplementary information. The clusters are consistent with those reported in the previous section for both influential journals and authors.

Regarding influential journals (Figure 4A), Science of the Total Environment, Environmental Pollution, Environmental Research, and Atmospheric Environment are key in the air quality cluster. The blue cluster includes journals such as Science, Nature, the Lancet, Proceedings of the National Academy of Sciences of the United States of America, The New England Journal of Medicine, and PLOS One, with a focus on key issues such as the transmission dynamics of the pandemic in cities, and environmental factors associated with the survival and spread of the virus. In the red cluster, three main sub-clusters could be identified: the sub-cluster on urban planning and urban studies is represented by journals such as Sustainable Cities and Society, Cities, Urban Studies, and Sustainability; the sub-cluster at the bottom includes journals such as Landscape and Urban Planning, Urban Forestry and Urban Greening, and Health and Place that can be linked to the issues related to urban green space and associated ecosystem services discussed earlier; finally, there are journals such as Transport Policy, Transportation Research Part A: Policy and Practice, and Journal of Transport Geography in the top-left corner that have published on transportation and travel behavior impacts.

As for influential authors (Figure 4B), most have published air quality changes as expected. These are scholars like Shubham Sharma (Indian Institute of Technology Delhi), Aurelio Tobías (Institute of Environmental Assessment and Water Research), and Susanta Mahato (University of Gour Banga), that have central positions in the green cluster. In the blue cluster on the impacts of pollution and other environmental factors on the exposure to and spread of the virus, key authors are Muhammad Farhan Bashir (Central South University), Yaron Ogen (Institute of Geosciences and Geography), and Yongjian Zhu (University of Science and Technology of China). Mario Coccia (National Research Council of Italy) and Jingui Xie (University of Science and Technology of China) also focus on similar issues. Other authors in the red cluster have published on other issues related to pandemic transmission and control: authors like Moritz Kraemer (University of Oxford), Matteo Chinazzi (Northeastern University), and Huaiyu Tian (Beijing Normal University) have published on the effects of human mobility and travel restrictions on the spread of the pandemic; works of scholars like Ayyoob Sharifi (Hiroshima University) and Naglaa Megahed (Port Said University) are related to lessons for urban planning and design; and key authors publishing on transportation and travel behavior are Jonas De Vos (University College London), Matthew Beck (The University of Sydney), and Alejandro Tirachini (Universidad de Chile).

Results of bibliographic coupling to identify countries that have made more contributions to research on cities and the COVID-19 pandemic show the dominance of the US, China, England, Canada, Spain, Japan, and Australia (Figure S1). Although developed countries have made more contributions, results show that the topic has also been studied in developing countries around the world. In fact, countries like Iran, Mexico, Malaysia, Chile, Pakistan, and Nigeria have made considerable contributions. This is a promising finding as it is projected that the majority of the future increase in the world’s urban population will occur in developing countries. Cumulatively, China, India, and Nigeria will account for 37% of this increase (UN, 2018). This provides an opportunity for better planning and design toward creating sustainable and resilient cities in the post-COVID era. The analysis also showed institutions that have made more contributions. Here, too, institutions from the US, China, and England are dominant (Figure S2 and Table S4).

### Conclusions

Cities worldwide have been the hotspots of the COVID-19 pandemic. The pandemic and its impacts on cities have allowed researchers to reflect on current urban planning, design, and management patterns to make positive transformations toward urban resilience and sustainability. This study aimed to provide an overview of the existing research on cities and the COVID-19 pandemic, identify major thematic areas, and highlight under-explored areas that need further research.

The bibliometric analysis of 2,799 articles published in 2020 and 2021 showed that multiple issues had been addressed in the literature. These were divided into six broad themes: air quality, environmental/meteorological factors, built environment factors, transportation, inequalities and socio-economic disparities, and smart city solutions and technologies. In 2020 and 2021, the themes of air quality, environmental/meteorological factors, and built environment factors are dominant. The main change in 2021 is that the themes of transportation and smart city solutions and technologies have expanded, indicating increasing research in these areas.

The list of the well-studied and under-explored areas is presented in Table 2. This overview showed that much research has been published on issues related to changes in urban air quality that can be used to inform urban environmental quality. A relatively under-explored area is impacts on CO_2_ and CH_4_ emissions in cities. Exploring such impacts is critical for addressing the looming issue of climate change. Furthermore, more research is needed to examine if the air quality changes have been temporary and what should be done to achieve permanent improvements.

**Table 2:** The list of the well-studied and under-explored areas

Theme	Well-studied areas	Under-explored areas
Air quality	• Impacts on the concentration levels of particulate matter, NO_2_, SO_2_, CO, and O_3_	• Impacts on CO_2_ and CH_4_ • Air quality after returning to normal
Environmental/meteorological factors	• Impacts of pollution on the transmission of the virus • Impacts of air temperature and humidity on the transmission of the virus • Impacts on water resources	• Impacts of wind on the transmission of the virus • Differential impacts on minorities and urban poor • Impacts on the waste sector
Built environment and urban planning	• Spatiotemporal spread patterns • Impacts of density • Restructuring and retrofit of public spaces • Infrastructure accessibility • Urban green spaces	• Dynamics and patterns of urban population loss and suburban sprawl • Urban-rural linkages • Urban governance issues
Socio-economic impacts	• Disproportionate impacts on minorities and urban poor • Health disparities • Supply chain disruptions	• Medium- and long-term impacts on the urban economy • Gender impacts
Transportation	• Impacts on public transportation • Travel behavior changes • Active transportation patterns	• Long-term impacts • Recovery of public transportation • Equitable access to alternative transport modes
Smart city solutions and technologies	• Contributions to pandemic control • Contributions to maintaining urban functionality	• Privacy and data security • Digital divide

The theme on environmental/meteorological factors has covered multiple issues related to the impacts of pollution on the transmission of the virus, impacts of air temperature and humidity on the transmission of the virus, and impacts on water resources. However, more research on the impacts of wind on the transmission of the virus, differential environmental impacts on minorities and urban poor, and pandemic impacts on the waste sector is needed. Addressing these gaps is essential for ensuring a green and inclusive recovery from the pandemic.

Much has been published regarding urban planning and built environment factors that indicate the importance of urban planning and non-pharmaceutical interventions in promoting human health. Spatiotemporal analyses of the spread patterns have contributed to a better understanding of the influential factors (Maiti et al., 2021). Issues such as adequate infrastructure accessibility, availability and distribution of green spaces, and restructuring and retrofit of open and public spaces have been particularly emphasized. A key issue that needs further research is the impact of the pandemic on population dynamics in cities. Given the increasing penetration of smart solutions that have increased teleworking and mobile work, it is likely that population dynamics in city regions will change, and counter-urban attitudes will result in relocation to suburbs. Exploring such changes and taking appropriate planning strategies is needed to ensure that they will not undermine the sustainability and resilience of city regions (e.g., by damaging fertile agricultural lands and ecosystems, increasing the carbon footprint of urban areas, building settlements on risk-prone areas, having negative impacts on local identity, exacerbating inequalities, and so forth). In some contexts, suburban development could be inevitable, as more people will work from home and the need for living in big cities may decline. Under such circumstances, it is essential to take regenerative urban design measures to reconfigure suburban areas and make them more compact and walkable (Moglia et al., 2021). Also, proper urban governance and regional planning strategies are needed to regulate urban-rural linkages. Such strategies are critical for maintaining ecosystem services and minimizing food supply chain disruptions during adverse events like pandemics, and need to be better explored (Blay-Palmer et al., 2021).

Existing research has further exposed urban inequalities and disproportional impacts on urban racial and ethnic minorities and low-income groups. However, impacts on some demographic groups in cities like children and women are less studied and warrant further research. As only two years have passed and some economic impacts take more time to appear, existing research on economic impacts has mainly focused on issues such as supply chain disruptions, and discussions on other impacts are mainly speculative and not empirically grounded. Therefore, more evidence-based research on medium- and long-term economic impacts on cities is needed. In the context of increasing urbanization and climate change, the investment choices we make today play an important role in shaping the future of cities. It is needed to study how the economic impacts of the pandemic have influenced the investment and expenditure choices of local government. This could provide insights to make sure that climate-resilient and sustainable activities are not derailed.

Multiple issues related to transportation and travel behavior have been studied. The pandemic has called into question the safety and desirability of existing urban transit patterns and has led to shifts in travel behavior. Public transit ridership has plummeted in many cities. Future research should examine if public transit systems can return to normal in the coming year and explore actions that need to be taken to enhance their safety. A major shift to active modes such as cycling has been observed in some cities. It is worth examining whether this will continue and what should be done to ensure equitable access to active transportation infrastructure.

Smart city solutions and technologies have also been widely studied in the context of the pandemic. Existing research has mainly focused on contributions to pandemic control and ensuring the continuity of urban activities. Although smart cities can enhance the capacity of cities to prepare for, absorb the shocks, and recover from adverse events like pandemics, more research on privacy and data security issues is needed. Additionally, the digital divide issue needs further exploration to ensure equitable access to urban services and opportunities.

Overall, this thematic analysis indicates that the pandemic is expected to have significant repercussions for life in cities. Now, about three years into the pandemic is the right time to take stock of the lessons learned to move toward integrating the principles of resilience and sustainability into urban planning, design, and management. This is essential for addressing the challenges of climate change, which is a more serious crisis looming over cities.

### Limitations of the study

This study has contributed to unpacking some of those repercussions. However, there are some limitations that need to be acknowledged. Bibliometric analysis is an effective method for providing an overview of a research field, identifying major thematic areas, and exploring the evolution of themes and concepts. However, to gain more comprehensive details, it should be complemented with systematic reviews. Therefore, systematic reviews on specific themes and sub-themes mentioned in this article are needed to understand the impacts of the pandemic better and provide more specific policy recommendations. Also, it should be mentioned that this study has only analyzed peer-reviewed research. Future research should also consider evidence reported in gray literature to ensure better coverage of real-world impacts and policy-focused activities that may not always be adequately reported in peer-reviewed academic literature.

## STAR★Methods

### Key resources table

**Table:** 

REAGENT or RESOURCE	SOURCE	IDENTIFIER
**Deposited data**		

Bibliographic details of peer-reviewed academic publications	Web of Science	https://www.webofscience.com/wos/woscc/basic-search

**Software and Algorithms**		

VOSviewer version 1.6.18	Centre for Science and Technology Studies, Leiden University	https://www.vosviewer.com/

### Resource availability

#### Lead contact

Further information and requests for resources should be directed to and will be fulfilled by the lead contact, Ayyoob Sharifi (sharifi@hiroshima-u.ac.jp).

#### Material availability

This study did not generate new materials.

### Method details

Any bibliometric analysis involves two major steps: creating a database of relevant articles and doing the analysis using existing software tools. More details related to these steps are presented here.

#### Database creation

Input data for bibliometric analysis is bibliometric data of documents indexed in academic databases. The first step in creating a bibliometric analysis database is delineating the review’s scope and developing a search string. As it is aimed to cover multiple issues related to cities and the pandemic, a broad-based search string was developed that is a combination of different terms related to COVID-19 and its impacts, cities, and urban planning, design, and management (Table S5).

This search string was developed in an iterative manner to ensure its appropriateness. In other words, outputs of an initial search string were screened to see if any missing terms could be added. This was repeated several times until adding new terms to the string did not yield more results (i.e., additional references). The Web of Science (WoS) was selected among different academic databases due to its reputation for indexing quality peer-reviewed literature. The PRISMA flowchart for literature identification, screening, and selection is shown in Figure S3 (Page et al., 2021).

Given the time lag between publication and indexing in the database, the literature search was conducted at the end of January 2022 to ensure the maximum inclusion of documents published in 2021. The literature search in the ‘title, abstract, and keywords’ field returned 6,232 records. Of these, 927 were excluded as they belonged to irrelevant WoS categories. The title and abstract of each record were checked manually to exclude irrelevant records. Inclusion criteria were: focusing on the COVID-19 pandemic and addressing urban-related issues such as impacts on cities and urban residents, transmission and control patterns and strategies in cities, lessons, and lessons and implications for urban planning, design, and management. As the search string was broad, it was found that many studies were irrelevant and should be excluded. For instance, many studies were focused on issues practiced in an urban context but irrelevant to urban planning, design, or management. Examples are studies focused on perceptions toward vaccination in specific cities (Crozier et al., 2021), or articles reporting issues/activities occurred in urban areas during the pandemic that are not related to urban planning and design (Curiel and Clark, 2021; Gavioli et al., 2022). After excluding irrelevant records, 2,799 remained in the database. Full record and citation details of these records were downloaded for bibliometric analysis.

#### Analysis using VOSviewer

Several software tools have been developed for more effective bibliometric analysis, following the advances in data analytics and text mining in the past two decades (Cobo et al., 2011b; Sharifi, 2021). These include tools such as SciMAT, CiteSpace, and VOSviewer. All these tools provide means for unpacking the complex interlinkages between different components of academic articles (i.e., keywords, journals, authors, cited references). However, there are differences between them in terms of the algorithms they use and their visualization styles (Cobo et al., 2011b). In this study, we have used VOSviewer because of its user-friendly interface and its relatively easy-to-interpret outputs and visualization. VOSviewer is a Java-based application that is freely available for download at: https://www.vosviewer.com/. The website also offers a comprehensive user manual with detailed explanations of different bibliometric analyses and their mathematical bases (Van Eck and Waltman, 2022). VOSviewer can be used to conduct different types of analyses. Here, we have used ‘term co-occurrence analysis’, ‘co-citation analysis’, and ‘bibliographic coupling’. These are briefly explained here. It is beyond the scope of this paper to provide details on the mathematical foundations of these analyses. Interested readers are referred to the VOSviewer manual for more detatils (Van Eck and Waltman, 2022).

The term co-occurrence analysis is used to identify key thematic areas and understand their interlinkages. The output of this analysis is a network of nodes and links, where node size is proportional to the number of times a term has co-occurred with other terms, and link thickness is proportional to the strength of the connection between two terms. Terms that co-occur frequently form a cluster that indicates a thematic research area. As different variants of a term may exist (e.g., ‘neighborhood’ and ‘neighbourhood’), before conducting the term co-occurrence analysis, a thesaurus file was developed and added to the VOSviewer database to ensure different variants are not considered separately. In addition to analyzing the whole database, separate analyses for 2020 and 2021 data were conducted to understand how the thematic focus has changed over time.

We used the co-citation analysis to identify the most influential journals, references, and authors. “A co-citation link is a link between two items that are both cited by the same document” (Van Eck and Waltman, 2022). A higher value of co-citation is argued to be an indicator of more influence (Van Eck and Waltman, 2022; Sharifi, 2021). “A bibliographic coupling link is a link between two items that both cite the same document” (Van Eck and Waltman, 2022). Bibliographic coupling is “another type of analysis that is commonly used to identify countries or institutions that have made more contributions to the development of a research field” (Van Eck and Waltman, 2022). Like the term co-occurrence analysis, the outputs of co-citation analysis and bibliographic coupling are presented as networks of nodes and links. Node size indicates the relative importance of the object in question (i.e., journals, references, authors, etc.); the thickness of a link connecting two nodes indicates the strength of the connection between them.

## Data Availability

Any additional information required to reanalyze the data reported in this paper is available from the lead contact upon request. This paper does not report original code.
