# Supplementary material for: An overview and thematic analysis of research on cities and the COVID-19 pandemic: Toward just, resilient, and sustainable urban planning and design
Source: iScience. 2022 Oct 7;25(11):105297. doi: 10.1016/j.isci.2022.105297 (PMC9540689; doi:10.1016/j.isci.2022.105297)
Supplement: Document S1. Figures S1–S3 and Tables S1–S5 [file mmc1.pdf]

iScience, Volume *25*

Supplemental information

An overview and thematic analysis of research on cities and the COVID-19 pandemic: Toward just, resilient, and sustainable urban planning and design

Ayyoob Sharifi

Table S1. Top 50 most influential references on cities and COVID-19, related to Figure 3.

Title	Source	Citations	Total link strength
Effect of restricted emissions during COVID-19 on air quality in India	Science of the Total Environment	172	1409
Changes in air quality during the lockdown in Barcelona (Spain) one month into the SARS-CoV-2 epidemic	Science of the Total Environment	159	1249
Effect of lockdown amid COVID-19 pandemic on air quality of the megacity Delhi, India	Science of the Total Environment	151	1211
The effect of human mobility and control measures on the COVID-19 epidemic in China	Science	139	411
COVID-19 pandemic and environmental pollution: A blessing in disguise?	Science of the Total Environment	121	959
The effect of travel restrictions on the spread of the 2019 novel coronavirus (COVID-19) outbreak	Science	118	331
The impact of COVID-19 partial lockdown on the air quality of the city of Rio de Janeiro, Brazil	Science of the Total Environment	116	1061
COVID-19 pandemic: Impacts on the air quality during the partial lockdown in São Paulo state, Brazil	Science of the Total Environment	112	1017
The COVID-19 pandemic: Impacts on cities and major lessons for urban planning, design, and management	Science of the Total Environment	111	227
Severe air pollution events not avoided by reduced anthropogenic activities during COVID-19 outbreak	Science of the Total Environment	110	704
An investigation of transmission control measures during the first 50 days of the COVID-19 epidemic in China	Science	95	347
Does lockdown reduce air pollution? Evidence from 44 cities in northern China	Science of the Total Environment	93	820
Lockdown for CoViD-2019 in Milan: What are the effects on air quality?	Science of the Total Environment	93	808
Indirect effects of COVID-19 on the environment	Science of the Total Environment	93	610
Aerosol and surface stability of HCoV-19 (SARS-CoV-2) compared to SARS-CoV-1	The New England Journal of Medicine	91	267
Association between short-term exposure to air pollution and COVID-19 infection: Evidence from China	Science of the Total Environment	88	611
Amplified ozone pollution in cities during the COVID-19 lockdown	Science of the Total Environment	87	735
The effect of COVID-19 and subsequent social distancing on travel behavior	Transportation Research	83	250

	Interdisciplinary Perspectives		
Assessing nitrogen dioxide (NO_2) levels as a contributing factor to coronavirus (COVID-19) fatality	Science of the Total Environment	83	612
Does Density Aggravate the COVID-19 Pandemic?	Journal of the American Planning Association	81	131
Assessing air quality changes in large cities during COVID-19 lockdowns: The impacts of traffic-free urban conditions in Almaty, Kazakhstan	Science of the Total Environment	78	780
Temporary reduction in daily global CO_2 emissions during the COVID-19 forced confinement	Nature Climate Change	78	374
Clinical features of patients infected with 2019 novel coronavirus in Wuhan, China	The Lancet	77	297
COVID-19 as a factor influencing air pollution?	Environmental Pollution	76	677
Impact of Coronavirus Outbreak on NO_2 Pollution Assessed Using TROPOMI and OMI Observations	Geophysical Research Letters	74	487
Changes in U.S. air pollution during the COVID-19 pandemic	Science of the Total Environment	71	576
Antivirus-built environment: Lessons learned from Covid-19 pandemic	Sustainable Cities and Society	71	163
Can atmospheric pollution be considered a co-factor in extremely high level of SARS-CoV-2 lethality in Northern Italy?	Environmental Pollution	69	450
COVID-19 lockdowns cause global air pollution declines	PNAS	69	522
A preliminary assessment of the impact of COVID-19 on environment – A case study of China	Science of the Total Environment	69	578
A Novel Coronavirus from Patients with Pneumonia in China, 2019	The New England Journal of Medicine	68	302
An interactive web-based dashboard to track COVID-19 in real time	The Lancet Infectious Diseases	67	111
Effects of temperature variation and humidity on the death of COVID-19 in Wuhan, China	Science of the Total Environment	65	350
The socio-economic implications of the coronavirus pandemic (COVID-19): A review	International Journal of Surgery	65	114
Air quality changes during the COVID-19 lockdown over the Yangtze River Delta Region: An insight into the impact of human activity pattern changes on air pollution variation	Science of the Total Environment	64	551

Correlation between weather and Covid-19 pandemic in Jakarta, Indonesia	Science of the Total Environment	64	428
The Management Transformation of Huawei<br>From Humble Beginnings to Global Leadership	Cambridge University Press	64	306
Effects of the COVID-19 Lockdown on Urban Mobility: Empirical Evidence from the City of Santander (Spain)	Sustainability	62	254
Association between ambient temperature and COVID-19 infection in 122 cities from China	Science of the Total Environment	62	340
Nowcasting and forecasting the potential domestic and international spread of the 2019-nCoV outbreak originating in Wuhan, China: a modelling study	The Lancet	61	177
Decline in PM 2.5 concentrations over major cities around the world associated with COVID-19	Environmental Research	58	525
Correlation between climate indicators and COVID-19 pandemic in New York, USA	Science of the Total Environment	56	410
Impact of Covid-19 lockdown on PM10, SO2 and NO2 concentrations in Salé City (Morocco)	Science of the Total Environment	56	524
A familial cluster of pneumonia associated with the 2019 novel coronavirus indicating person-to-person transmission: a study of a family cluster	The Lancet	55	165
Role of the chronic air pollution levels in the Covid-19 outbreak risk in Italy	Environmental Pollution	55	397
Modal share changes due to COVID-19: The case of Budapest	Transportation Research Interdisciplinary Perspectives	54	252
Unexpected air pollution with marked emission reductions during the COVID-19 outbreak in China	Science	54	374
The short-term impacts of COVID-19 lockdown on urban air pollution in China	Nature Sustainability	53	379
Early Transmission Dynamics in Wuhan, China, of Novel Coronavirus–Infected Pneumonia	The New England Journal of Medicine	53	121
COVID-19 and Public Transportation: Current Assessment, Prospects, and Research Needs	Journal of Public Transportation	53	124

Table S2. Top 20 most influential journals contributing to the literature on cities and COVID-19, related to Figure 4.

Source	Citations	Total link strength
Science of the Total Environment	5393	132029
Sustainability	1731	34953
Lancet	1422	30504
International Journal of Environmental Research and Public Health	1270	32803
Atmospheric Environment	1229	42203
Science	1127	25606
Plos One	982	20508
Sustainable Cities and Society	954	25301
Atmospheric Chemistry and Physics	883	31127
Proceedings of the National Academy of Sciences of the United States of America	856	21589
Environmental Pollution	850	30786
Nature	827	18800
Environmental Research	784	26949
The New England Journal of Medicine	678	14399
Landscape and Urban Planning	609	16767
The Journal of the American Medical Association	589	10511
Cities	585	11700
Journal of Cleaner Production	578	14388
Aerosol and Air Quality Research	577	20977
Transport Policy	558	14387

Table S3. Top 20 most influential authors contributing to the literature on cities and COVID-19, related to Figure 4.

**Author**	**Citations**	**Total link strength**
Sharma, S	190	1579
Tobias, A	186	1457
Mahato, S	163	1335
Sharifi, A	155	404
Kraemer, Mug	150	459
Venter, ZS	138	665
Hamidi, S	131	211
Muhammad, S	122	1034
Nakada, LYK	121	1109
Coccia, M	120	634
Wang, PF	120	843
Chinazzi, M	119	375
Dantas, G	118	1102
Kumar, P	118	888
Sicard, P	117	850
Wang, Q	113	796
Zambrano-monserrate, MA	112	814
Collivignarelli, MC	107	909
Wu, X	107	511
Liu, Y	105	346

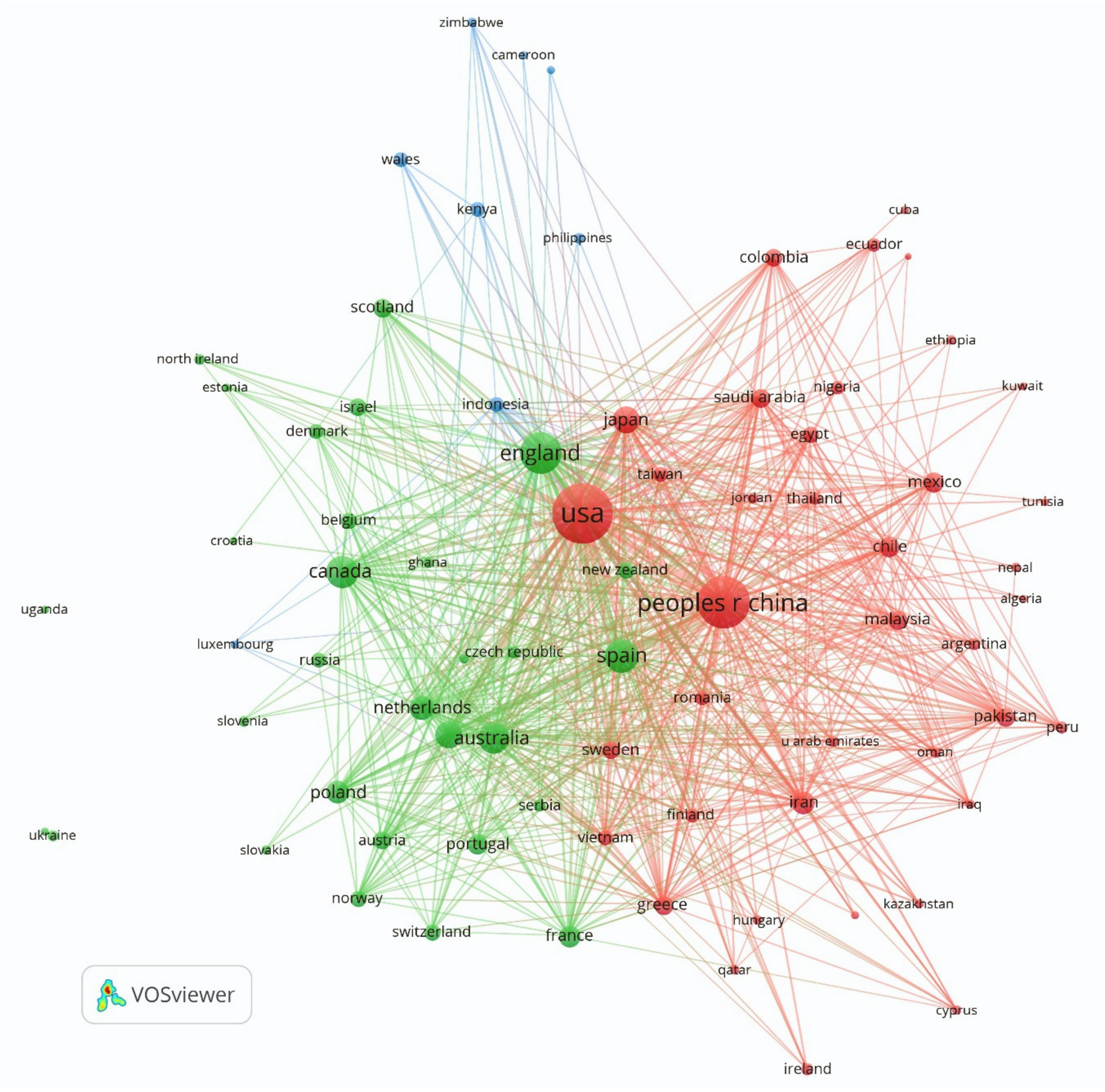

Figure S1. Countries that have contributed more to research on COVID-19 and cities. Larger node size indicates more contribution to the literature and link thickness is proportional to the strength of the connection between two countries. Countries are divided into three clusters based on their level of research collaboration.

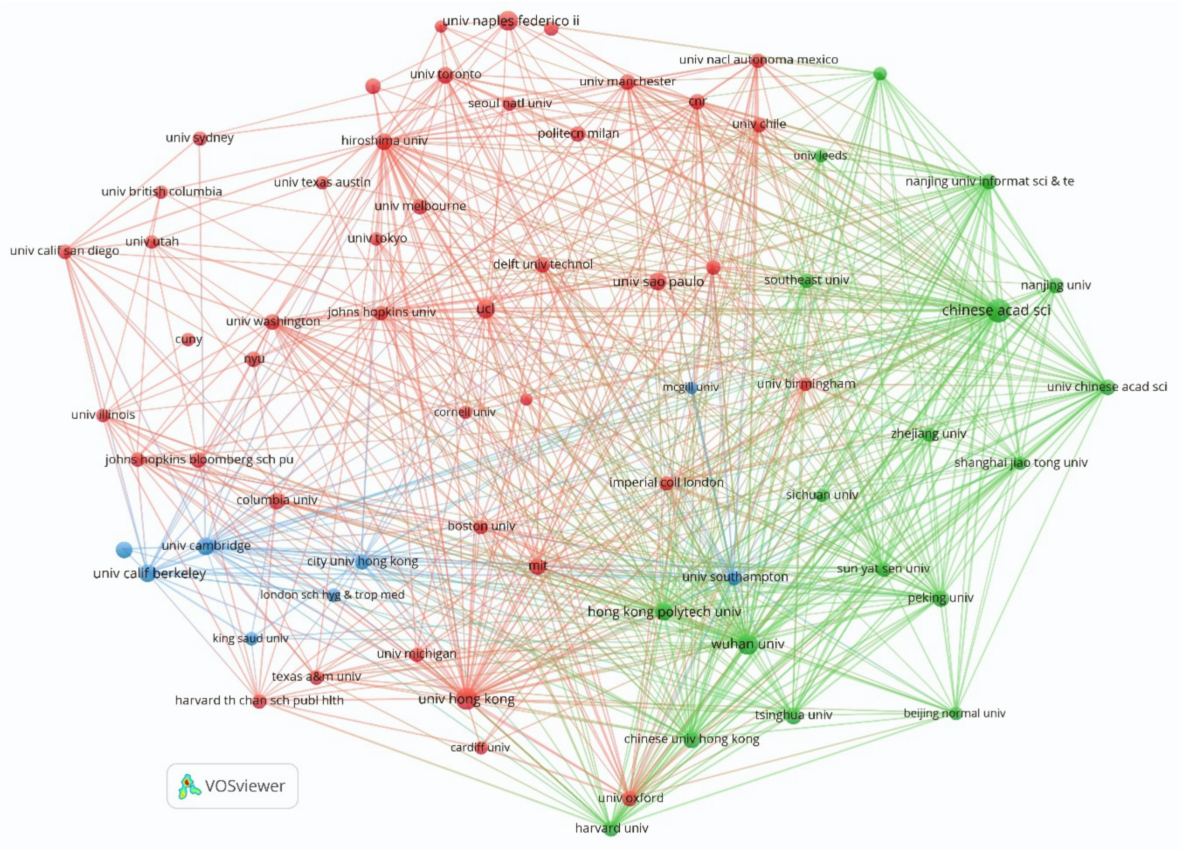

Figure S2. Institutions that have contributed more to research on COVID-19 and cities, related to Table S4. Larger node size indicates more contribution to the literature and link thickness is proportional to the strength of the connection between two institutions. Institutions are divided into three clusters based on their level of research collaboration.

Table S4. List of major institutions contributing to the literature on cities and COVID-19, related to Figure S2.

**Organization**	**Documents**	**Citations**	**Total link strength**
University of Hong Kong	38	2841	6968
University of Oxford	16	1002	3612
Harvard University	16	960	5224
Peking University	21	943	5677
Tsinghua University	20	869	4592
University of Southampton	14	821	4366
Beijing Normal University	11	765	3105
Chinese Academy of Sciences	50	720	13191
National Research Council (Italy)	17	554	3517
University of Cambridge	22	467	3989

Table S5. The search string, related to STAR Methods.

1	TS=((("covid*" OR "coronavirus" OR “corona” OR "pandemic*" OR “SARS-CoV-2” OR “Global Health Crisis” OR “epidemic*”) AND (“urban plan*” OR “spatial plan*” OR “urban design*” OR “city plan*” OR “town plan*” OR “urban manage*” OR “neighbo*rhood plan*” OR “urban neighbo*rhood*” OR “neighbo*rhood design” OR “*minute cit*“ OR “*minute neighbo*rhood*” OR “urbanism” OR “urban studies” OR “urbanist*” OR “sustainable cit*” OR “sustainable urban” OR “urban sustainab*” OR “resilient cit*” OR “urban resilien*” OR “post-pandemic cit*” OR “post pandemic cit*” OR “post-corona cit*” OR “post corona cit*” OR “post-covid cit*” OR “post covid cit*” OR “smart cit*” OR “smart technolog*” OR “smart solution*” OR “future of cit*” OR “urban future*” OR “urban lifestyle” OR “urban place*” OR “Urban space*” OR “urban experience*” OR “urban life” OR “urban liv*” OR “compact cit*” OR “compact urban” OR “urban compact” OR “urban sprawl” OR “urban growth” OR “urban expansion” OR “suburban development” OR “suburb*” OR “peri-urban” OR “peri urban” OR “population density” OR “residential density” OR “city size” OR “outdoor space*” OR “public space*” OR “street reallocation” OR “street re-allocation” OR “street design” OR “built environment*” OR “built areas” OR “built-up areas” OR “urban-rural” OR “urban rural” OR “rural-urban” OR “rural urban” OR “public transport*” OR “public transit*” OR “active transport*” OR “park use” OR “park visit” OR “access to open space*” OR “access to outside space*” OR “residential preference*” OR “residential choice*” OR “residential perception*” OR “urban perception*” OR “urban preference*” OR “tourist* cit*” OR “urban infrastructure” OR “greening of cities” OR “bike-sharing”)))
2	TS=((("covid*" OR "coronavirus" OR “corona” OR "pandemic*" OR “SARS-CoV-2” OR “Global Health Crisis” OR “epidemic*”) AND ((“urban*” OR “city” OR “cities” OR “built environment*” OR “neighbo*rhood*” OR “town*” Or “district*” Or “metropoli*”) NEAR/8 (“spatial disparit*” OR “open space*” OR “socio-economic” OR “density” OR “street” OR “transport*” OR “transit*” OR “mobilit*” OR “bicycle” OR “bike” OR “cycling” OR “pedestrian*” OR “social impact*” OR “equit*” OR “poverty” OR “poor” OR “inequalit*” OR “marginaliz*” OR “justice” OR “minorit*” OR “refugee*” OR “homeless” OR “immigrant*” OR “income” OR “park” OR “preference*” OR “choice*” OR “perception*” OR “preference*” OR “climat*” OR “environment*” OR “pollution” OR “air” OR “water” OR “econom*” OR “business” OR “supply chain” OR “touris*” OR “green*” OR “recovery” OR “governance” OR “nature” OR “health” OR “forest*” OR “ecosystem” OR “food” OR “agricult*” OR “vulnerabilit*” OR “services” OR“emission*” OR “resident*”))))
3	#2 OR #1 and 2021 or 2022 or 2020 (Publication Years)

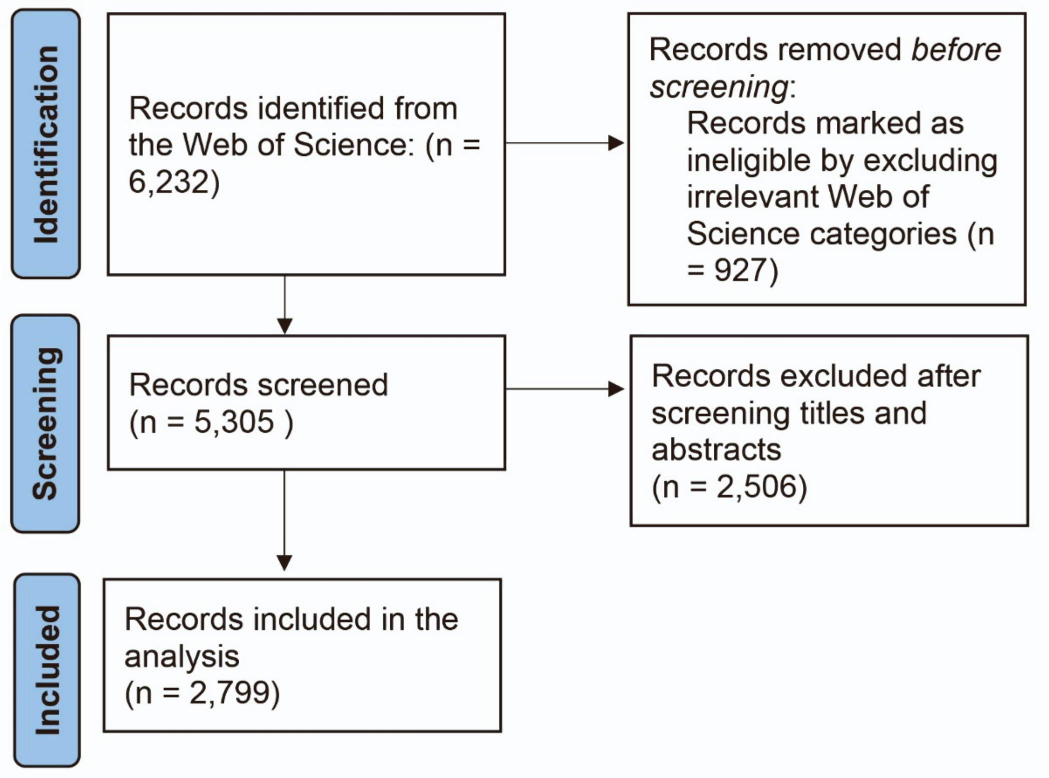

Figure S3. The PRISMA flowchart for literature identification, screening, and selection (Adapted from Page et al. (2021)), related to STAR Methods.

References

Page, M. J., McKenzie, J. E., Bossuyt, P. M., Boutron, I., Hoffmann, T. C., Mulrow, C. D., Shamseer, L., Tetzlaff, J. M., Akl, E. A., Brennan, S. E., Chou, R., Glanville, J., Grimshaw, J. M., Hróbjartsson, A., Lalu, M. M., Li, T., Loder, E. W., Mayo-Wilson, E., McDonald, S., . . . Moher, D. (2021). The PRISMA 2020 statement: an updated guideline for reporting systematic reviews. *BMJ*, *372*, n71. https://doi.org/10.1136/bmj.n71
